# SIRT2 knockout exacerbates insulin resistance in high fat-fed mice

**DOI:** 10.1371/journal.pone.0208634

**Published:** 2018-12-11

**Authors:** Louise Lantier, Ashley S. Williams, Curtis C. Hughey, Deanna P. Bracy, Freyja D. James, Muhammad A. Ansari, David Gius, David H. Wasserman

**Affiliations:** 1 Department of Molecular Physiology & Biophysics, Vanderbilt University School of Medicine, Nashville TN, United States of America; 2 Vanderbilt Mouse Metabolic Phenotyping Center, Nashville, TN, United States of America; 3 Department of Radiation Oncology, Northwestern University Feinberg School of Medicine, Chicago, IL, United States of America; Université catholique de Louvain, BELGIUM

## Abstract

The NAD^+^-dependent deacetylase SIRT2 is unique amongst sirtuins as it is effective in the cytosol, as well as the mitochondria. Defining the role of cytosolic acetylation state in specific tissues is difficult since even physiological effects at the whole body level are unknown. We hypothesized that genetic SIRT2 knockout (KO) would lead to impaired insulin action, and that this impairment would be worsened in HF fed mice. Insulin sensitivity was tested using the hyperinsulinemic-euglycemic clamp in SIRT2 KO mice and WT littermates. SIRT2 KO mice exhibited reduced skeletal muscle insulin-induced glucose uptake compared to lean WT mice, and this impairment was exacerbated in HF SIRT2 KO mice. Liver insulin sensitivity was unaffected in lean SIRT2 KO mice. However, the insulin resistance that accompanies HF-feeding was worsened in SIRT2 KO mice. It was notable that the effects of SIRT2 KO were largely disassociated from cytosolic acetylation state, but were closely linked to acetylation state in the mitochondria. SIRT2 KO led to an increase in body weight that was due to increased food intake in HF fed mice. In summary, SIRT2 deletion *in vivo* reduces muscle insulin sensitivity and contributes to liver insulin resistance by a mechanism that is unrelated to cytosolic acetylation state. Mitochondrial acetylation state and changes in feeding behavior that result in increased body weight correspond to the deleterious effects of SIRT2 KO on insulin action.

## Introduction

SIRT2 is unique in that it is the only sirtuin known to exist in both the mitochondria and cytosol [1]. Like all members of the sirtuin family, SIRT2 is NAD^+^-dependent [2]. SIRT2 links lysine acetylation to cellular energy homeostasis, as this reaction is regulated by both acetyl-CoA availability and NAD^+^ levels. In accordance with this, protein acetylation is determined by nutritional and metabolic states [3, 4]. The first demonstrations of enzyme acetylation regulating flux through metabolic pathways sparked great interest in this protein modification [5, 6]. Overnutrition and obesity lead to increased lysine acetylation as a consequence of increased acetyl-CoA [7–10]. This has led to the hypothesis that protein hyperacetylation contributes to the pathogenesis of insulin resistance and type 2 diabetes [11].

Recent studies have specifically focused on the link between glucose homeostasis and mitochondrial protein acetylation, providing evidence that these events are coupled [4, 8, 12]. The link between increased mitochondrial protein acetylation and impaired glucose metabolism is particularly striking during overnutrition, as various models of muscle mitochondrial hyperacetylation show impaired glucose metabolism [9, 10, 13]. In stark contrast, the impact of cytosolic protein acetylation on glucose metabolism *in vivo* is unknown. In the present study, genetic and dietary means were used to study the effect of increased acetylation state. Mice with a whole-body knockout (KO) of SIRT2 and their wildtype (WT) littermates were fed a chow or high fat (HF) diet. A whole-body KO was used as a first step as potential sites of action have not been defined and this will provide us with the opportunity to do so. The hypothesis tested was that loss of SIRT2 would induce hyperacetylation and impaired insulin sensitivity. It was further hypothesized that the insulin resistance due to diet-induce obesity would be exacerbated with deletion of the SIRT2 gene.

## Research design and methods

### Mouse models

**Fig 1:**
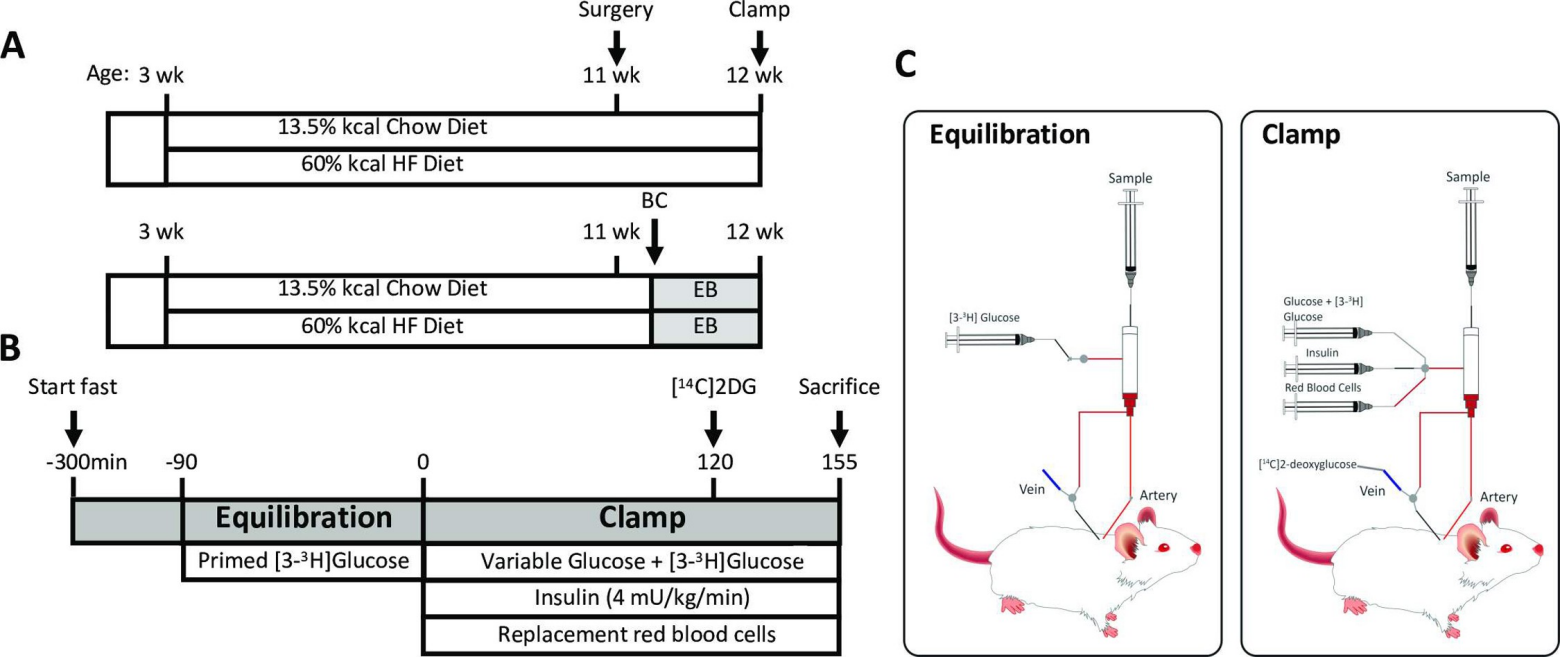
Study design and insulin clamp protocol for the conscious unrestrained mouse. **A**: All mice were randomized to either chow or HF diet at 3 weeks of age. Studies were performed at 12 weeks of age. BC: body composition; EB: energy balance. **B**: Insulin clamp protocol. **C**: Insulin clamp setup. Mice are never handled nor retrained after they are hooked up to the swivel (t = -90min). [^14^C]2DG: 2[^14^C]deoxyglucose.

Male mice lacking the SIRT2 protein (SIRT2 KO) and their wild type littermates (WT) on a C57BL/6J background [14] were fed a chow (13.5% calories from fat; 5001, LabDiet) or HF diet (60% calories from fat; F3282, BioServ) for 9 weeks beginning at 3 weeks of age (Fig 1A). Mice were housed in a temperature/humidity-controlled environment with a 12h light cycle. All studies were performed on 12 week 5h-fasted male mice. The Vanderbilt Animal Care and Use Committee approved all animal procedures specifically for this study. Animals were anesthetized by pentobarbital injection and sacrificed as approved by the Vanderbilt Animal Care and Use Committee.

### Hyperinsulinemic euglycemic clamp (insulin clamp)

Catheters were surgically placed in the carotid artery and jugular vein for sampling and infusions, respectively, one week before insulin clamps were performed. Mice were fasted for 5h before clamps (Fig 1B and 1C). Mice were neither restrained nor handled during clamp experiments [15]. Erythrocytes were infused to compensate for blood withdrawal. [3-^3^H]glucose was primed and continuously infused from *t* = -90min to *t* = 0min (0.04 μCi/min). The insulin clamp was initiated at *t* = 0min with a continuous insulin infusion (4 mU/(kg·min)) and variable glucose infusion was maintained until t = 155min. The glucose infusate contained [3-^3^H]glucose (0.06μCi/μL) to minimize changes in [3-^3^H]glucose specific activity. Arterial glucose was monitored every 10 min to provide feedback to adjust the glucose infusion rate (GIR) as needed to clamp arterial glucose concentration. [3-^3^H]glucose kinetics were determined at -15min and -5min for the basal period, and for the clamp period every 10min between 80 and 120min. A 13μCi intravenous bolus of 2[^14^C]deoxyglucose ([^14^C]2DG) was administered at 120min to determine the glucose metabolic index (R_g_), a measure of tissue-specific glucose uptake. Blood samples were collected at 122, 125, 135, 145 and 155min to measure [^14^C]2DG disappearance from plasma. Tissues were freeze-clamped to assess tissue-specific R_g_ and perform subsequent analyses. A full step-by-step description of the surgery, isotope clamp method and calculations is available from the Vanderbilt Mouse Metabolic Phenotyping Center (MMPC) web site (www.vmmpc.org). A weight-matched subgroup of HF mice was formed by excluding mice whose body weight was greater than 1 standard deviation from the mean body weight of all clamped HF-fed mice (mean ± SD criteria: 38.4 ± 4.2 grams, leading to group sizes of 7 WT and 10 KO mice, see Table 1).

**Table 1:** Characteristics of the clamped WT and SIRT2 KO mice on a chow or HF diet.

	Chow Diet		HF Diet		Weight-matched HF Diet Subgroups	
	WT	SIRT2 KO	WT	SIRT2 KO	WT	SIRT2 KO
**N**	7	9	12	13	7	10
**Body Weight (g)**	24.9±0.9	27.2±0.7	36.5±1.5 ^#^	39.5±1.0 ^#^	37.9±1.4 ^#^	39.5±0.8 ^#^
**Fasting blood glucose** **(mg/dL)**	125 ± 5	128 ± 7	154 ± 7 ^#^	166 ± 7 ^#^	163 ± 8 ^#^	171 ± 8 ^#^
**Clamp blood glucose (mg/dL)**	151 ± 4	156 ± 5	154 ± 2	155 ± 2	154 ± 1	154 ± 2
**Fasting Insulin (ng/mL)**	1.5 ± 0.2	1.4 ± 0.2	4.1 ± 0.7 ^#^	6.3 ± 0.9 ^#^	4.2 ± 0.6 ^#^	5.6 ± 0.6 ^#^
**Clamp Insulin (ng/mL)**	6.7 ± 1.1	5.8 ± 0.7	11.8 ± 1.7	12.1 ± 1.6	12.2 ± 2.1	11.9 ± 1.6
**GIR (mg/kg/min)**	53 ± 4	45 ± 3	32 ± 3	25 ± 2 *	28.5 ± 3.7	25.5 ± 1.9
**Fasting endoRa (mg/kg/min)**	18.3 ± 0.7	16.9 ± 1.1	14 ± 0.6	13 ± 0.3	13.7 ± 0.5	12.7 ± 0.3
**Clamp endoRa (mg/kg/min)**	1.7 ± 1.2	6.6 ± 2.1	2 ± 1	7 ± 0.9 **	3.3 ± 1.1	6 ± 0.9
**Clamp Rd (mg/kg/min)**	55.1 ± 3.5	50.0 ± 2.8	35 ± 2.8	31 ± 1.7	31.8 ± 2.9	31.6 ± 2.2
**% endoRa Suppression**	90 ± 7	62 ± 12	83 ± 7	50 ± 6 **	76 ± 8	53 ± 7
**Fasting plasma leptin** **(ng/mL)**	0.8 ± 0.2	0.5 ± 0.2	37.3±9.0 ^#^	40.1 ± 5.9 ^#^	37.1 ± 8.9 ^#^	37.1 ± 5.9 ^#^

The weight-matched subgroup of HF mice includes mice whose body weight was within 1 Standard Deviation of the mean body weight of all clamped HF mice (i.e. 38.4 ± 4.2 grams).

* p<0.05;

** p<0.01 KO vs. WT within the same diet.

^#^ p<0.05 HF vs. Chow within the same genotype.

GIR: Glucose Infusion Rate; endoRa: endogenous Rate of glucose appearance; Rd: Rate of glucose disappearance.

### Plasma and tissue tracer processing

Radioactivity of [3-^3^H]glucose, [^14^C]2DG and [^14^C]2DG-6-phosphate were determined as previously described [16], as well as whole-body glucose appearance (R_a_) endogenous glucose production (endoR_a_; a measure of hepatic glucose production), and tissue-specific glucose uptake R_g_ [17, 18]. Glycogen was determined using the method of Chan and Exton [19]. Tissue triglycerides were measured by an enzyme-linked spectrophotometric assay (Triglycerides-GPO reagent set, Pointe Scientific). Plasma insulin were determined by ELISA (Millipore). Tissue acetyl-CoA levels were determined by fluorescent enzymatic assay (Abcam 87546). Citrate synthase activity was assessed enzymatically as previously described [9].

### Energy balance

Body composition was measured by NMR. Energy expenditure was measured using indirect calorimetry (Promethion, Sable Systems). Mice were individually placed in metabolic cages with bedding (identical to home-cages) within the Vanderbilt MMPC in a 12h light cycle, temperature/humidity-controlled room for 5 days. Data represent the last 4 days of measurement. Cage air is sampled through microperforated stainless steel tubes located at the bottom of the cages ensuring that cage air is sampled uniformly. Promethion utilizes a pull-mode, negative pressure system with an excurrent flow rate of 2L/min. Water vapor is continuously measured and its dilution effect on O_2_ and CO_2_ are mathematically compensated for during data analysis [20]. O_2_ consumption and CO_2_ production are measured for each mouse for 30sec every 5min. Room air reference gases are sampled every 4 cages. The respiratory quotient (RQ) is the ratio of CO_2_ production over O_2_ consumption. Energy expenditure is calculated using the Weir equation [21]. Ambulatory activity is determined every second with XYZ beams. Data acquisition and raw data processing were coordinated by MetaScreen v2.2.18 and ExpeData v1.7.30.

### Tissue protein lysates and Western blots

After either a 5h fast or an insulin clamp, mice were anesthetized and tissues were flash-frozen. Mitochondrial and cytosolic protein fractions were obtained as previously described [22]. Whole tissue lysates were obtained by homogenization in Lysis Buffer containing 25mM Tris-HCl pH7.4, 10mM EDTA, 10% Glycerol, 1% Triton-X100, HALT protease/phosphatase inhibitor cocktail (Pierce) and deacetylase inhibitors nicotinamide (10mM) and Trichostatin A (1μM), then centrifuged at 13,000 rpm for 10 min at 4°C. 20μg of the supernatant was applied to 4–12% SDS-PAGE. Immunoblots were incubated with primary antibodies against acetylated-lysine (Cell Signaling 9814; 1:500), phospho-Akt (Ser473) (Cell Signaling 9271; 1:1000), Akt (Cell Signaling 9272; 1:1000), phospho-IRS1 (Ser302) (Cell Signaling 2491; 1:1000), IRS1 (Cell Signaling 2382; 1:1000), phospho-FOXO1 (Ser256) (Cell Signaling 9461; 1:1000), FOXO1 (Cell Signaling 2880; 1;1000), SIRT3 (Cell Signaling 54905; 1:1000), SIRT2 (Proteintech 196551AP; 1:600). Equal loading was assessed by blotting for β-actin, VDAC or GAPDH (Abcam 8224, 15895, and 9484 respectively, 1:1000). Secondary antibodies (Li-Cor or Cell Signaling, 1:10,000) were incubated at room temperature for 1h and visualization and quantification were performed using the Odyssey imaging system (Li-Cor) or chemiluminescence. All gels included samples from each group. A total n = 6–8 mice per group were analyzed for each protein. Intensities were normalized to WT within each blot.

### Real time PCR

mRNA was extracted from freeze-clamped livers using the Qiagen RNeasy Mini kit, and gene expression was determined with the following Taqman Assays (ThermoFisher) according to manufacturer’s instructions: adgre (F4/80) (Mm00802529_m1), il6 (Mm00446190_m1) and il1b (Mm00434228_m1). Results were analyzed using the 2^-ΔΔCt^ method with GAPDH (Mm99999915_g1) as reference gene [23].

### Statistics

Data are expressed as mean±SE. Data points outside of average±1.5*SD were considered outliers and excluded from analysis. Statistical analyses were performed using two-tailed unpaired Student’s t-test (for two group analysis) or two-way factorial ANOVA for independent factors, followed by Tukey’s post hoc test for four group analysis (* symbols referring to genotype effect, and # symbols referring to diet effect). Energy expenditure was analyzed using the Analysis of Covariance (ANCOVA) by regressing energy expenditure (kcal/hr) against body weight [24]. ANCOVA-adjusted values for EE were calculated using the ANCOVA-generated regression equation. The relationship between body weight and GIR was similarly tested by the ANCOVA. The ANCOVA analysis was provided by the MMPC Energy Expenditure Analysis website (http://www.mmpc.org/shared/regression.aspx). The significance level for all tests was p<0.05 (*p<0.05; **p<0.01; ***p<0.001). @ p<0.05 WT vs. SIRT2 KO by ANCOVA when regression is run against body weight.

## Results

### SIRT2 deletion increases weight gain

**Fig 2:**
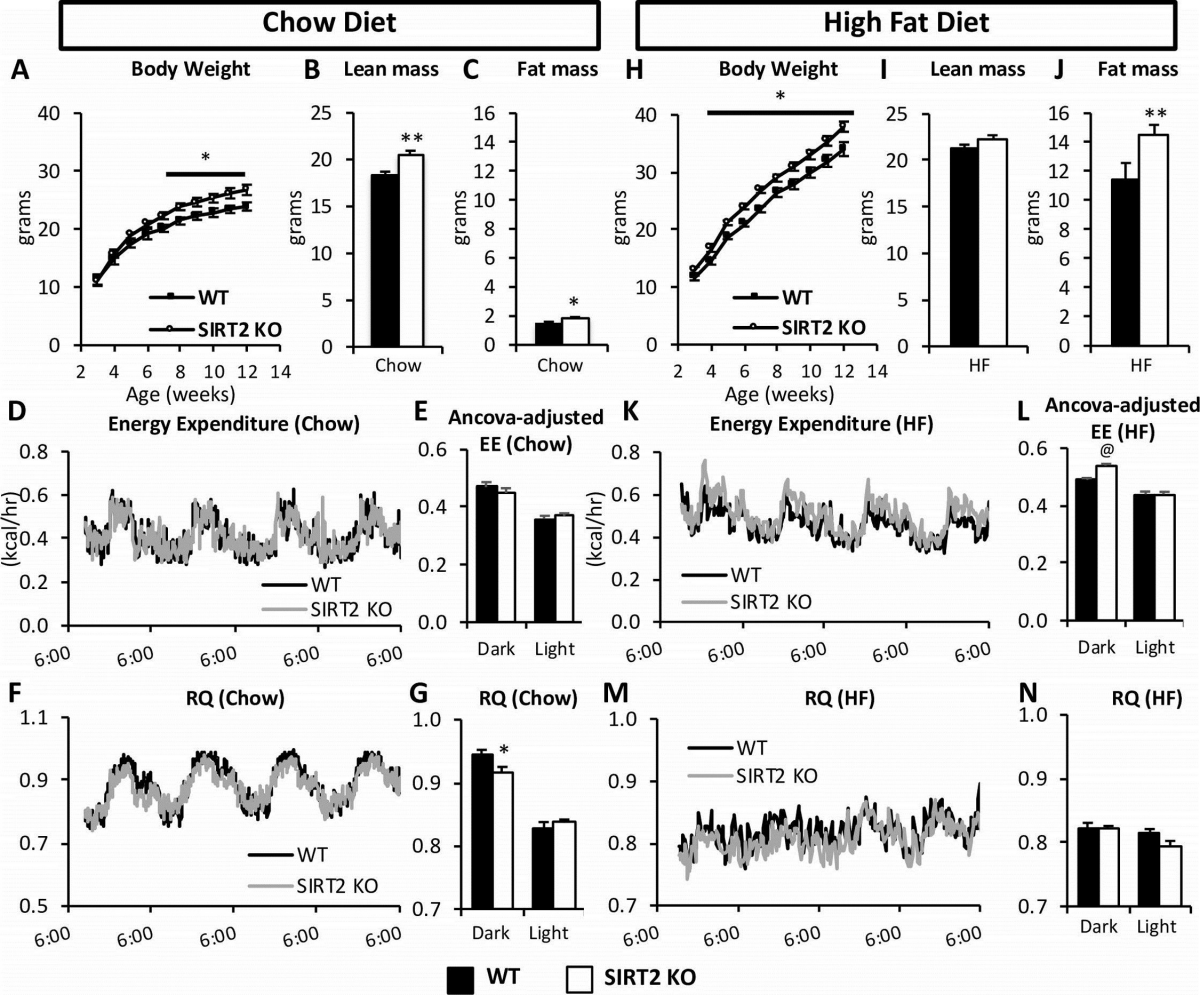
HF SIRT2 KO mice exhibit increased adiposity and energy expenditure. (**A**) Body weight of the chow WT and SIRT2 KO mice. Lean (**B**) and fat mass (**C**) in WT and SIRT2 KO mice on a chow diet at 12 weeks of age. Energy Expenditure (EE) over time (**D**) or ANCOVA-adjusted 12h average of EE (**E**) in chow-fed WT or SIRT2 KO mice. RQ over time (**F**) or expressed as 12h averages (**G**) in chow-fed WT or SIRT2 KO mice. **(H)** Body weight of the HF-fed WT and SIRT2 KO mice. Lean (**I**) and fat mass (**J**) in WT and SIRT2 KO mice on a HF diet at 12 weeks of age. Energy Expenditure (EE) over time (**K**) or ANCOVA-adjusted 12h average of EE, regressed to body weight (**L**) in HF-fed WT or SIRT2 KO mice. RQ over time (**M**) or expressed as 12h averages (**N**) in HF-fed WT or SIRT2 KO mice. @ p<0.05 WT vs. SIRT2 KO by ANCOVA when regression is run against body weight, absolute fat mass or absolute fat-free mass. There was no interaction between body weight, lean mass, fat mass, and Energy Expenditure. (n = 8/group for all panels). Black bars: WT; open bars: SIRT2 KO.

To investigate the interaction between SIRT2 deletion and diet, we monitored weight gain in WT and SIRT2 KO mice during 9 weeks of chow or HF diet, after which body composition was assessed and mice were placed in an indirect calorimetry system (Fig 1A). Chow-fed SIRT2 KO mice exhibited greater weight gain (Fig 2A) which was due to an increase in lean and fat mass (Fig 2B and 2C). Energy expenditure (EE) was unchanged, and this was confirmed by the ANCOVA analysis (Fig 2D and 2E). The respiratory quotient (RQ) was decreased in the dark phase (Fig 2F and 2G). On a HF diet, SIRT2 KO mice had increased weight gain starting at 4 weeks of age (Fig 2H) and fat mass was significantly increased at 12 weeks of age (Fig 2I and 2J). EE was slightly higher in SIRT2 KO mice during the dark cycle (Fig 2K and 2L). RQ was not different between genotypes on a HF diet (Fig 2M and 2N). Whole body deletion of SIRT2 was assessed by Western blot (S1A Fig). SIRT2 protein levels were unchanged by HF-feeding in skeletal muscle or liver (S1B Fig). SIRT3 is an important regulator of mitochondrial protein acetylation, and changes in its expression could induce changes in the mitochondrial acetylome. However neither gastrocnemius nor liver SIRT3 protein expression were altered with either diet or SIRT2 deletion (S1C and S1D Fig). Gastrocnemius acetyl-CoA was significantly increased in HF SIRT2 KO relative to chow SIRT2 KO mice (S1E Fig), but liver acetyl-CoA was unchanged by diet or genotype (S1F Fig). Citrate synthase activity, an index of mitochondrial content, was unchanged in gastrocnemius or liver (S1G and S1H Fig).

**Fig 3:**
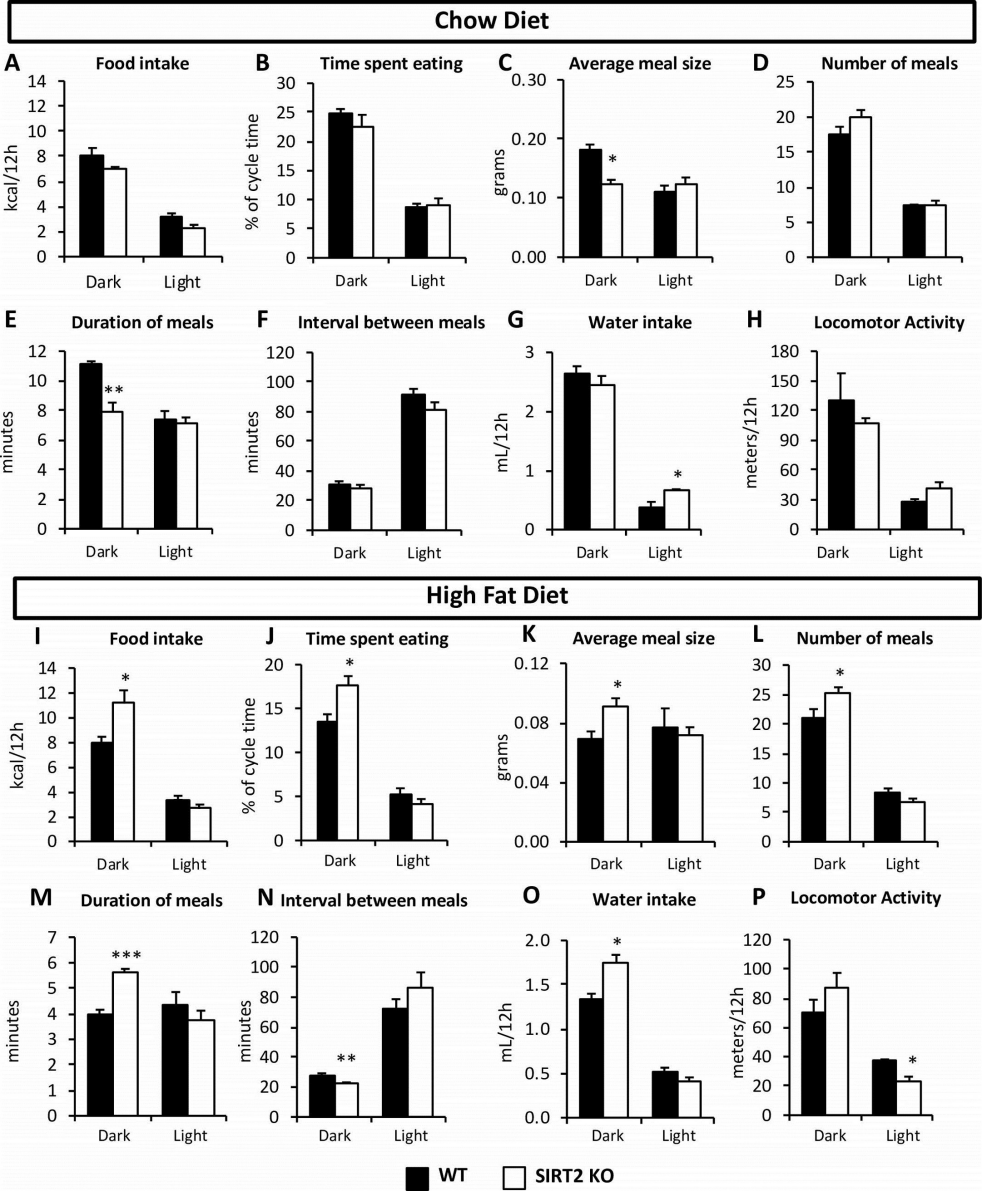
Feeding behavior was altered in HF SIRT2 KO mice. Total food intake in kcal per 12h cycle (**A, I**), time spent eating per 12h cycle (**B, J**), average individual meal size (**C, K**), number of meals per 12h cycle (**D, L**), average individual meal duration (**E, M**) and average time interval between meals (**F, N**) in chow and HF-fed mice, respectively, during either dark or light cycle (average of four days). Water intake (**G, O**) and locomotor activity (**H, P**) in chow or HF-fed mice respectively, during either dark or light cycle (average of four days). (n = 8/group for all panels). Black bars: WT; open bars: SIRT2 KO.

The increased fat mass of the HF SIRT2 KO mice occurred because food intake was increased by ~40% in these mice (Fig 3I). Feeding behavior in HF SIRT2 KO mice was altered: these mice ate larger, longer meals, with more frequency when compared to HF WT mice (Fig 3J–3N). These alterations in food intake were not observed in chow-fed mice (Fig 3A). The feeding behavior itself (i.e. meal patterning and feeding habits) was significantly different, but this was without consequences on the overall amount of kcal ingested. Because the SIRT2 KO mice had shorter meals (Fig 3E), but ate more often (Fig 3D), the time spent eating, as % of cycle time, was not altered between the WT and SIRT2 KO mice on chow diet (Fig 3B). Therefore, the difference in individual meal size did not manifest itself in overall calorie intake in the chow-fed mice. Investigation of whole brain protein acetylation levels showed that the acetylation status of what appears to be a single protein with a molecular weight of ~40kDa was distinctly and significantly regulated by SIRT2, as its acetylation status was increased 4-fold in the SIRT2 KO mice (S2 Fig). Other visible proteins were unaltered by either genotype or diet.

### SIRT2 KO mice have decreased skeletal muscle insulin-stimulated glucose uptake

**Fig 4:**
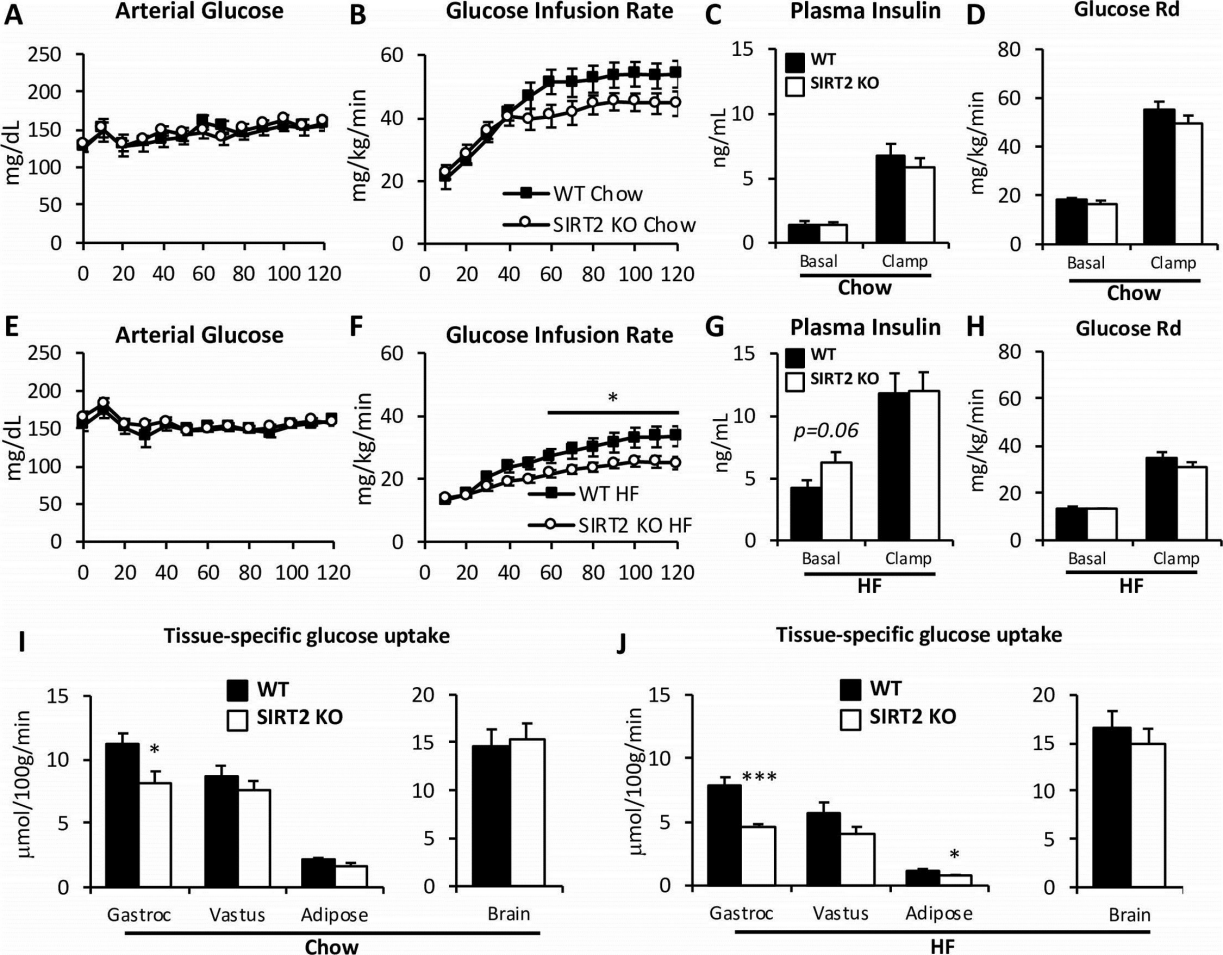
SIRT2 KO mice exhibit muscle insulin resistance during the hyperinsulinemic-euglycemic clamp. **A, E**: Blood glucose was monitored throughout the clamp at 10-min intervals by sampling from the arterial catheter. Blood glucose was maintained at euglycemia (∼150 mg/dL) in both chow-fed (**A**) and HF-fed (**E**) mice. The GIR in the venous catheter needed to maintain euglycemia in chow-fed (B) or HF-fed (**F**) mice. Plasma insulin levels at baseline and during the clamp in chow (**C**) or HF-fed mice (**G**). Rd, rate of glucose disappearance, in chow-fed (**D**) or HF-fed (**H**) mice, determined by administration of [3-3H]glucose. Rg in gastrocnemius, vastus lateralis, white epidydimal adipose tissue, and brain is determined by the administration of nonmetabolizable 2[14C]deoxyglucose in chow-fed (**I**) or HF-fed (**J**) WT and SIRT2 KO mice. (n = 7-13/group, see Table 1). Black bars: WT; open bars: SIRT2 KO. For clamp data of weight-matched HF subgroup, see Table 1 and Fig 8.

**Fig 5:**
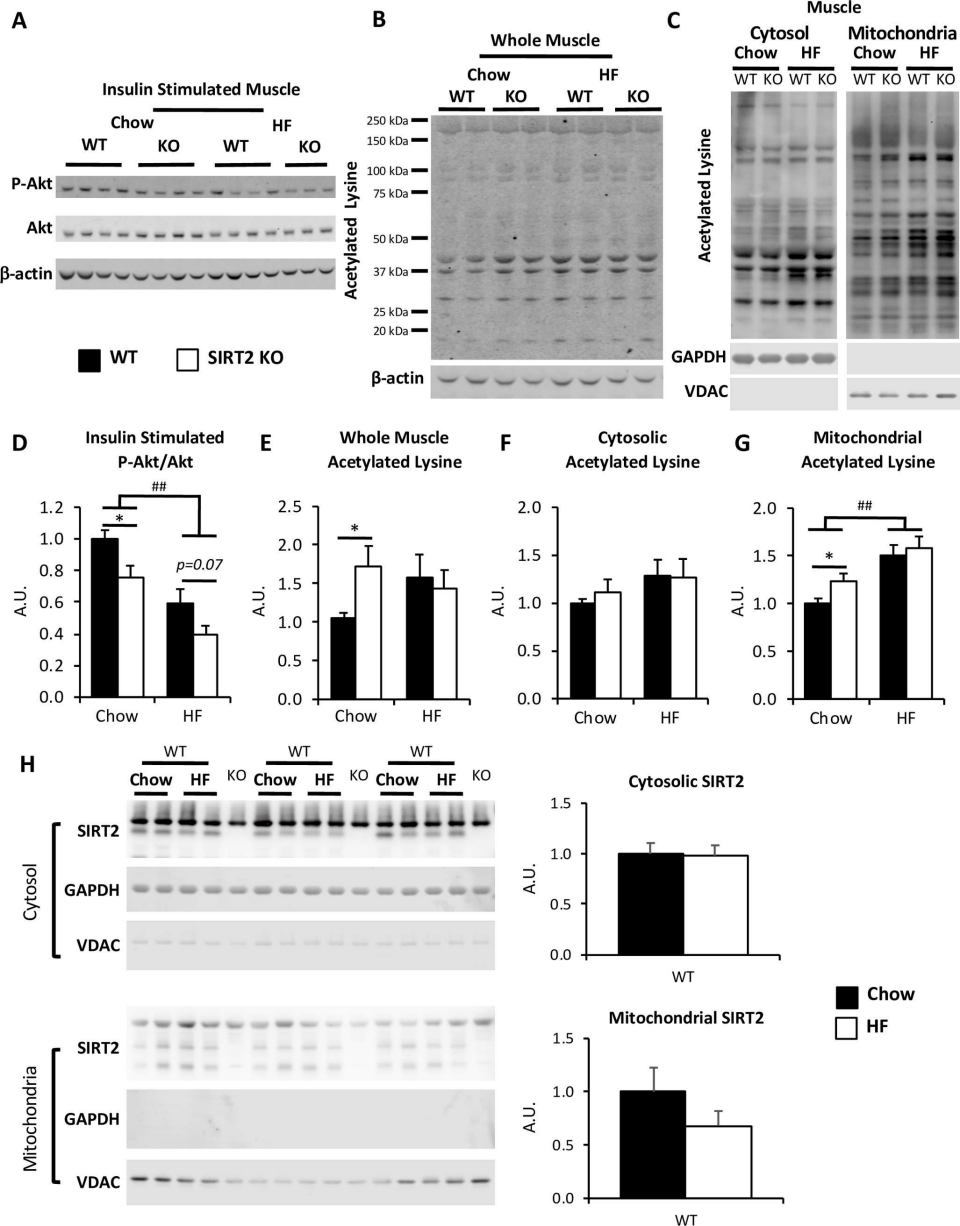
Insulin-induced skeletal muscle Akt phosphorylation were reduced in SIRT2 KO mice. **A, D**: Immunoblots for P-Akt (Ser473), Akt, and β-actin on gastrocnemius homogenates from chow-fed and HF-fed insulin clamped mice. Integrated intensities for P-Akt were obtained by the Odyssey software and normalized to Akt intensities (n = 7/group). **B, E**: Immunoblots for acetylated lysine and β-actin on gastrocnemius homogenates from chow-fed and HF-fed insulin clamped mice. Integrated intensities were obtained by the Odyssey software and normalized to β-actin intensities (n = 8/group). **C, F, G**: Immunoblots for acetylated lysine, GAPDH and VDAC on cytosolic or mitochondrial gastrocnemius homogenates. Integrated intensities (F, G) were obtained by the Odyssey software and normalized to GAPDH or VDAC respectively (n = 6/group). Black bars: WT; open bars: SIRT2 KO. **H**: Immunoblots for SIRT2 in cytosolic (top) and mitochondrial (bottom) protein fractions extracted from gastrocnemius muscle from 5h-fasted WT and SIRT2 KO mice on either a chow of HF diet. SIRT2 intensities were normalized to GAPDH (cytosolic fraction) or VDAC (mitochondrial fraction) (n = 6/group). Black bars: Chow; open bars: HF.

Fasting arterial glucose was not different between WT and SIRT2 KO mice (Fig 4A and 4E). However, HF-feeding significantly increased fasting arterial glucose in both genotypes (Fig 4 and Table 1). WT and SIRT2 KO mice underwent insulin clamps after either 9 weeks of chow or HF diets (Fig 1A). Fasting insulin was similar between WT and SIRT2 KO mice (Fig 4C and 4G). During the clamp, blood glucose was maintained at 150 mg/dL in all groups (Fig 4A and 4E). Insulin was increased equally in WT and SIRT2 KO littermates (Fig 4C and 4G). In HF-fed mice, the GIR was significantly reduced in SIRT2 KO compared to the WT mice, indicating greater insulin resistance in the HF-fed SIRT2 KO mice (Fig 4F). The clamp glucose disappearance (R_d_) was similar between genotypes (Fig 4H). Interestingly, glucose disappearance was not different between chow-fed SIRT2 KO and WT mice, but differences in R_g_ were observed in specific tissues. R_g_ was significantly reduced in gastrocnemius of chow SIRT2 KO mice compared to WT mice (Fig 4I). This reduction was further worsened in HF SIRT2 KO mice compared to HF WT littermates (Fig 4J). HF SIRT2 KO mice also exhibited a blunted white adipose tissue R_g_ (Fig 4J). Consistent with decreased gastrocnemius R_g_ in SIRT2 KO mice was a decrease in insulin signaling. SIRT2 KO mice on a chow diet exhibited a reduction in P-Akt/Akt (Thr473) compared to WT. HF diet induced a reduction in the ratio of P-Akt/Akt in muscle of WT mice, which was accentuated by SIRT2 KO (Fig 5A and 5D). P-IRS1/IRS (Ser302) and P-mTOR/mTOR (Ser2448) ratios were unchanged by diet or genotype (S3 Fig).

To test whether the defective muscle R_g_ in SIRT2 KO mice was associated with high protein acetylation we probed for acetylated lysine in muscle protein lysates. HF diet did not significantly increase lysine acetylation in whole gastrocnemius of WT mice (Fig 5B and 5E). Mitochondria of the same muscle exhibited a robust increase in mitochondrial protein acetylation, without an effect on cytosolic protein acetylation (Fig 5C, 5F and 5G). Deletion of SIRT2 in chow-fed mice significantly increased whole gastrocnemius lysine acetylation. However, lysine acetylation levels in gastrocnemius of HF SIRT2 KO mice were not further increased (Fig 5E). Probing for acetylated proteins in muscle cytosolic or mitochondrial fractions revealed that neither SIRT2 deletion nor HF diet significantly increased acetylation in muscle cytosol. This was in contrast to the increases observed in muscle mitochondria (Fig 5C, 5F and 5G). Neither cytosolic nor mitochondrial protein acetylation appeared to be altered in the vastus lateralis muscle (S4 Fig). To investigate these unexpected changes in gastrocnemius mitochondrial protein acetylation, we examined whether SIRT2 localization within the cell was altered with HF diet. While we were able to detect low levels of SIRT2 in the mitochondria of gastrocnemius, neither cytosolic nor mitochondrial SIRT2 protein levels were altered by HF diet in skeletal muscle (Fig 5H).

### HF-fed SIRT2 KO mice have exacerbated hepatic insulin resistance

**Fig 6:**
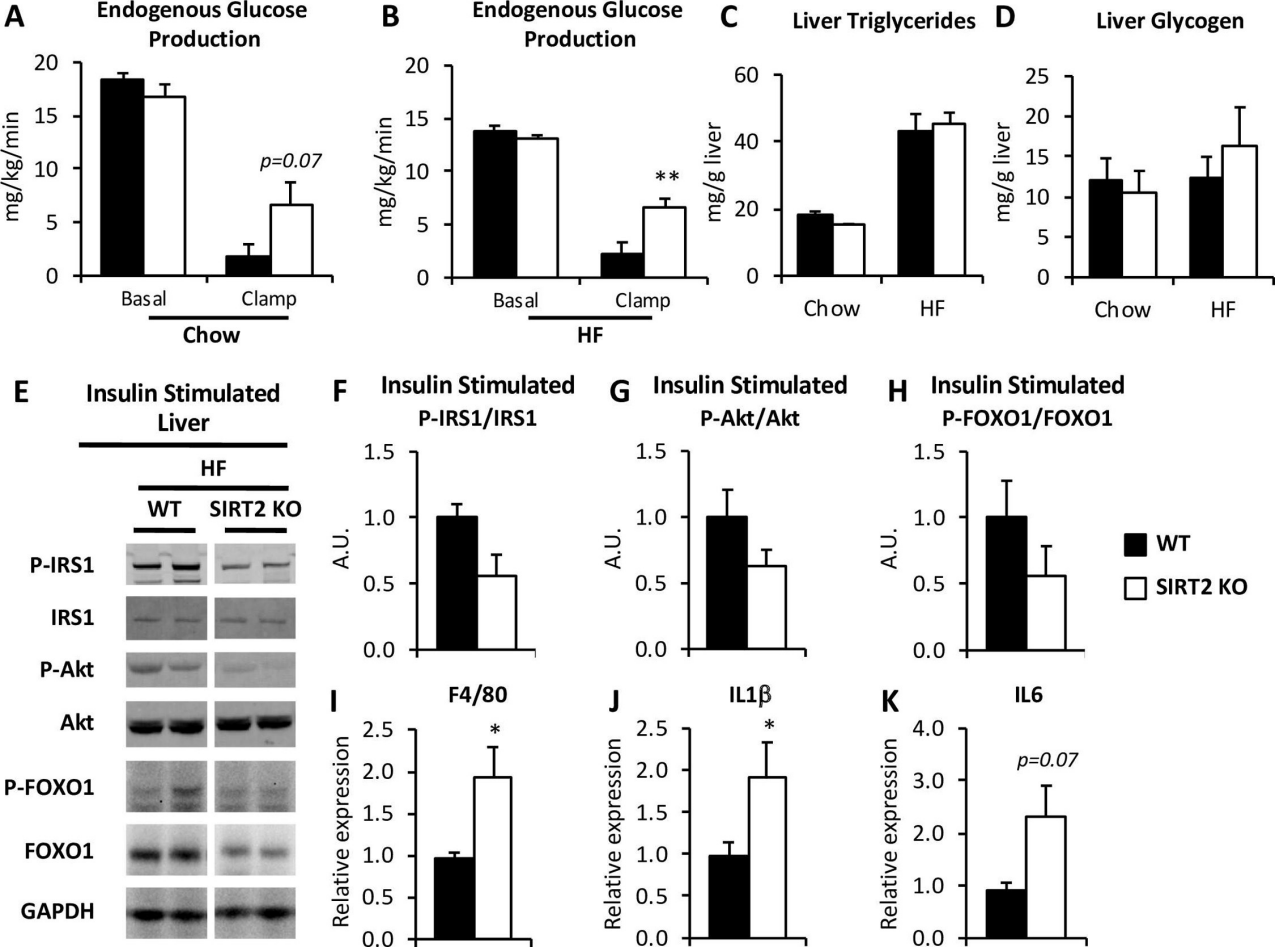
HF SIRT2 KO mice exhibit hepatic insulin resistance. **A, B**: endoRa, a marker of hepatic glucose production, in chow-fed (A) or HF-fed (B) mice, determined by administration of [3-3H]glucose during the insulin clamp (n = 7-13/group, see Table 1). **C, D:** Liver triglycerides (**C**) and liver glycogen (**D**) in chow and HF WT and SIRT2 KO mice (n = 7/group). **E**: Immunoblots for P-IRS1 (Ser302), IRS1, P-Akt (Ser473), Akt, P-FOXO1 (Ser256), FOXO1 and GAPDH on liver homogenates from clamped mice (n = 7/group). The uncropped Western blot is presented in S5 Fig. **F, G, H**: Integrated intensities were obtained by the Odyssey software and phospho-proteins were normalized to their respective total protein intensities (n = 7/group). **I, J, K**: Hepatic mRNA relative expression for adgre (F4/80) (**I**), il1b (**J**), il6 (**K**) from HF WT and SIRT2 KO mice (n = 8/group). Black bars: WT; open bars: SIRT2 KO.

EndoR_a_ was not different between genotypes in chow-fed mice in the basal state (Fig 6A). While insulin suppression of endoR_a_ tended to be reduced in chow-fed SIRT2 KO mice, rates were not significantly different compared to WT mice. In HF-fed mice, suppression of endoR_a_ was significantly reduced during the insulin clamp in SIRT2 KO mice compared to WT mice (Fig 6B). This reflects a further deterioration of liver insulin sensitivity in HF-fed SIRT2 KO mice.

The etiology of the liver insulin resistance observed in HF-fed mice was examined. Hepatic insulin resistance is generally associated with increased liver triglycerides. However, liver triglyceride content was not different between genotypes (Fig 6C). Liver glycogen was also unchanged between genotypes (Fig 6D). We performed immunoblotting on liver protein extracts from the insulin-clamped HF mice and assessed the phosphorylation status of three key proteins of the insulin signaling pathway: Insulin Receptor Substrate 1 (IRS1), Akt, and Forkhead box protein O1 (FOXO1). Differences in the phosphorylation to total ratios of these proteins was not different in HF-fed SIRT2 KO mice compared to HF-fed WT mice (Fig 6E–6H).

HF diet induces low-grade chronic inflammation and this has been proposed to be central to the pathogenesis of insulin resistance [25]. The expression of the pro-inflammatory genes encoding F4/80 and IL1β were significantly increased in the livers of HF-fed SIRT2 KO mice compared to HF-fed WT mice, and a similar trend was observed for the gene encoding IL6 (Fig 6I–6K). Overall these findings indicate a greater pro-inflammatory state in HF-fed SIRT2 KO livers.

### Liver protein acetylation is strongly increased in SIRT2 KO mice

**Fig 7:**
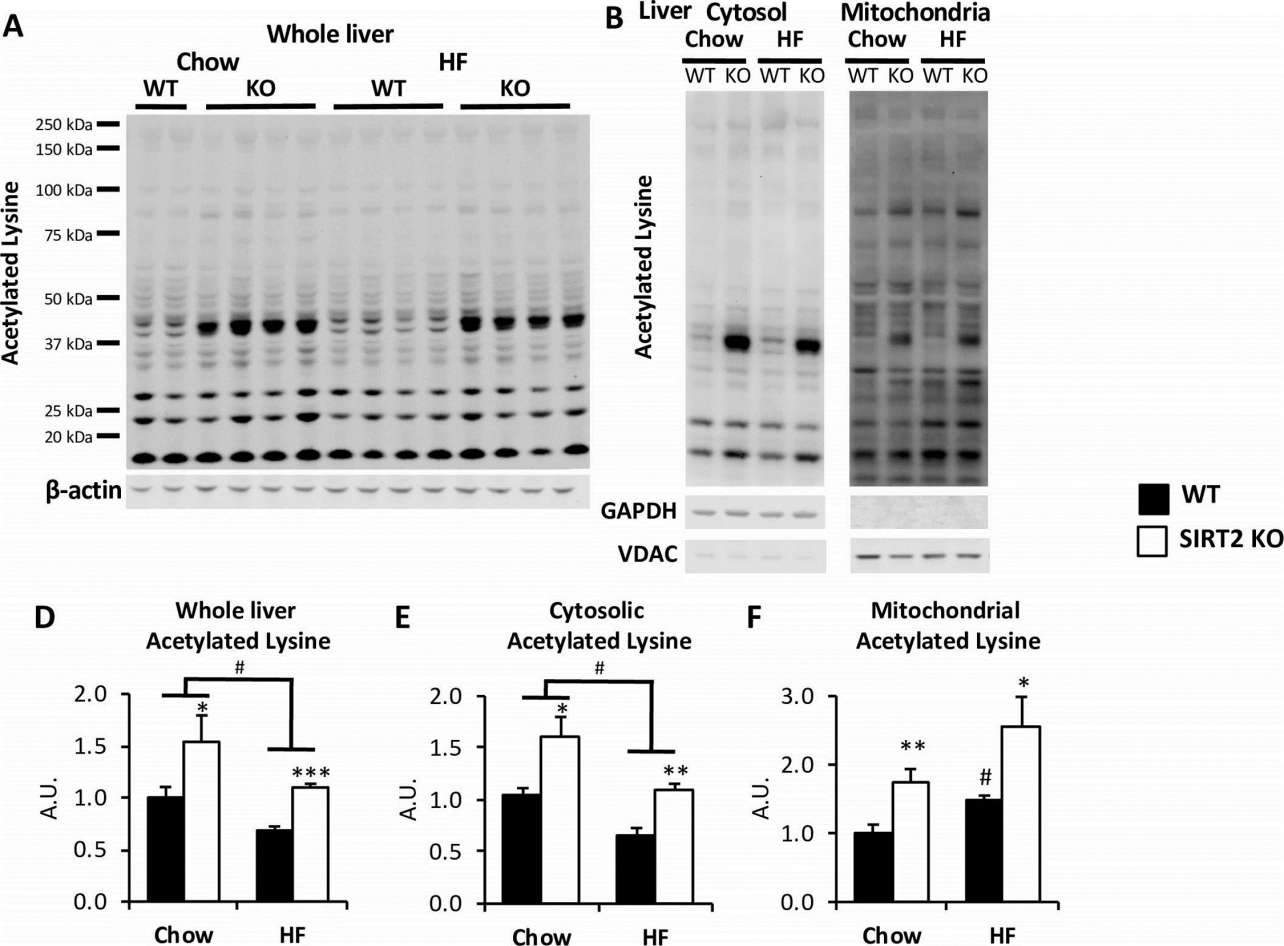
SIRT2 KO livers exhibit increased protein acetylation in both cytosolic and mitochondrial fractions. **A, B**. Immunoblots for acetylated lysine on whole liver (**A**) or cytosolic and mitochondrial liver fractions (**B**). Integrated intensities (**D, E, F**) were obtained by the Odyssey software and normalized to β-actin, GAPDH or VDAC intensities, respectively (n = 6/group).

We assessed the level of acetylated proteins in whole liver, cytosolic, and mitochondrial lysates (Fig 7A and 7B). SIRT2 KO caused liver protein hyperacetylation, independent of diet (Fig 7D), which appeared to be driven by robust hyperacetylation of a protein of a MW of ~40kDa. A band of the same size was also the most SIRT2-dependent acetylated protein in whole brain lysates (S2 Fig). HF-feeding caused a surprising reduction in protein acetylation in whole liver lysates, regardless of genotype (Fig 7D). The decrease in liver acetylation was due to reduced cytosolic acetylation, as mitochondrial protein acetylation was increased (Fig 7B, 7E and 7F). HF-fed SIRT2 KO mice showed increased liver acetylation in both cytosolic and mitochondrial compartments compared to the HF-fed WT mice. Despite the increase in cytosolic acetylation in HF SIRT2 KO mice compared to their HF WT littermates, it still remained below cytosolic acetylation of the chow-fed SIRT2 KO mice.

### The increased body weight of the HF SIRT2 KO mice is a determinant of their hepatic insulin resistance

**Fig 8:**
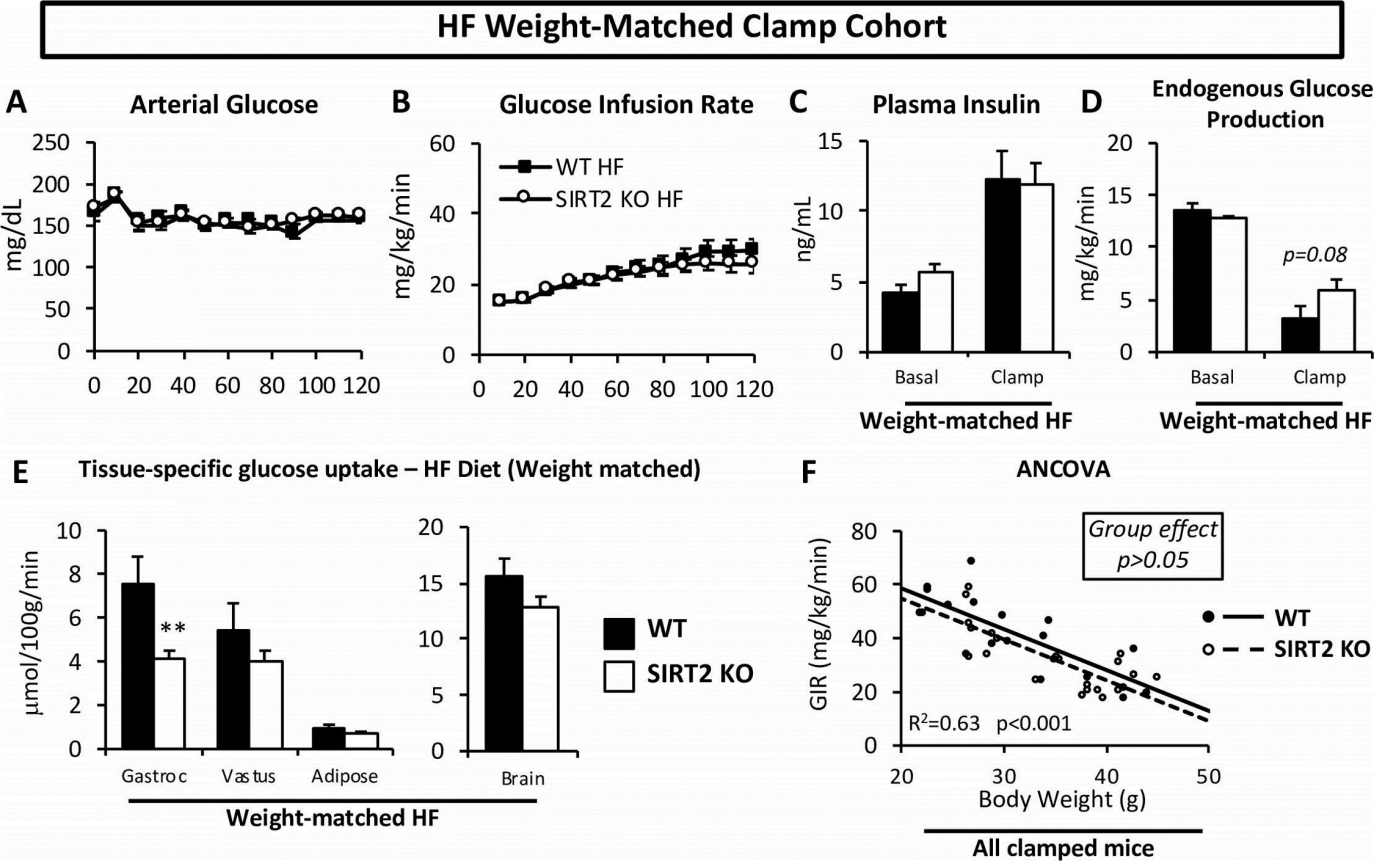
Weight-matched HF WT and SIRT2 KO mice only exhibit increased muscle insulin resistance during the insulin clamp. Mice whose body weights were within 1SD of the average body weight of all HF mice were included (38.4 ± 4.2 grams). Body weights of weight-matched HF subgroup are presented in Table 1. **A**: Blood glucose was monitored throughout the clamp at 10-min intervals by sampling from the arterial catheter. Blood glucose was maintained at euglycemia (∼150 mg/dL). **B**: The GIR in the venous catheter needed to maintain euglycemia in weight-matched HF-fed mice. **C**: Plasma insulin levels at baseline and during the clamp in weight-matched HF-fed mice. **D**: endoRa, a marker of hepatic glucose production, in weight-matched HF-fed mice, determined by administration of [3-3H]glucose. **E**: Rg in gastrocnemius, vastus lateralis, white epidydimal adipose tissue and brain is determined by the administration of nonmetabolizable glucose 2[14C]deoxyglucose in weight-matched HF-fed mice. **F**: GIR plotted against body weight for all WT and SIRT2 KO clamped mice (chow and HF). The regression lines were generated by the ANCOVA statistical tool. For clamp data of entire HF groups, see Figs 4 and 6 and Table 1 (n = 7-10/group for all panels, see Table 1).

Given the differences in weight and fat mass between HF-fed WT and SIRT2 KO mice, and that adiposity is a strong determinant of insulin sensitivity, we further examined the clamp phenotype using a weight-matched subgroup of HF-fed mice (Fig 8 and Table 1). This allowed us to determine whether differences observed in the clamp data were driven by the increase in body weight. The weight-matched subgroups were assembled by excluding mice with total body weights that were beyond 1 standard deviation of the average body weight of HF-fed mice, regardless of genotype. The weight-matched subgroup did not exhibit significantly different GIR between HF-fed WT and SIRT2 KO mice (Fig 8B and Table 1), suggesting that the additional weight gain was required for the whole-body insulin resistance in the HF-fed SIRT2 KO mice. EndoR_a_ was not significantly different between the weight-matched subgroups, showing that the greater hepatic insulin resistance was dependent on the increase in body weight in the HF-fed SIRT2 KO mice (Fig 8D). Interestingly, the reduced glucose uptake in gastrocnemius remained present in the weight-matched subgroup (Fig 8E). When GIR was plotted against body weight for all chow-fed or HF-fed mice (Fig 8F) we observed a significant correlation between GIR and body weight (r^2^ = 0.63, p<0.001), confirming that body weight is a strong determinant of GIR in the cohorts studied. Further, we showed that this relationship was not different between the two genotypes. Importantly, the ANCOVA linear model concluded that the two regression lines were not significantly different from each other (group effect, p = 0.16). This indicates that while the gastrocnemius resistance to insulin in SIRT2 KO mice was weight independent, whole body insulin action between SIRT2 KO and WT mice was similar for a given weight (Fig 8F).

## Discussion

SIRT2 is unique amongst sirtuins in that it was recently shown to be compartmentalized not only in the cytosol but also in the mitochondria [1]. A strong body of work has shown that mitochondrial acetylation is associated with metabolic disease [9, 13, 26]. The role of cytosolic acetylation in glucose homeostasis has received far less attention, even though the cytosol is the compartment where much of glucose metabolism (e.g. glycolysis, glucose production, glycogen synthesis) is regulated and the fact that cytosolic proteins undergo reversible acetylation [27]. Further, SIRT2 has been shown to be downregulated in visceral white adipose from obese subjects [28] as well as liver from ob/ob mice [29], suggesting SIRT2 may play an important role in the development of insulin resistance. In this study we used the whole body SIRT2 KO mouse to investigate the role of hyperacetylation on insulin action *in vivo*. The whole body KO is an appropriate first step since it allows for a global assessment of tissues involved. Moreover, a genetic mutation in patients leading to decreased expression of the SIRT2 gene would be present in the whole body. We hypothesized that deletion of SIRT2 would lead to increased acetylation and a more severe insulin resistance when fed a HF diet.

Lean SIRT2 KO mice exhibited insulin resistance in skeletal muscle. This was evidenced by a significant reduction in muscle R_g_ in insulin-stimulated conditions and a downregulation of Akt phosphorylation. Concomitant with this, protein acetylation levels in muscle were significantly increased in chow SIRT2 KO mice, showing that SIRT2 deletion is sufficient to induce robust hyperacetylation. Surprisingly, acetylation within the mitochondrial, but not cytosolic, compartment appeared to be the major driver for this finding, given that cytosolic acetylation was unaffected by either diet or SIRT2 deletion. Nevertheless, the absence of increases in the acetylation of cytosolic critical glucoregulatory proteins–without a detectable increase in overall cytosolic acetylation–cannot be ruled out in the HF SIRT2 KO muscle. Interestingly, a downregulation of the insulin-dependent phosphorylation cascade may not be a critical driver of the observed insulin resistance, since we did not observe significant downregulation of P-IRS1 or P-mTOR. However, we can postulate that acetylation events within the pathway may be altered, thereby altering pathway activity.

Interestingly, the increased mitochondrial acetylation found specifically in HF gastrocnemius was consistent with the increased acetyl-CoA in these tissues and the hypothesis that mitochondrial protein acetylation is subject to regulation by mass action [8]. The mitochondrial effect was also in agreement with a recent study that demonstrated the mitochondrial localization of SIRT2 and that absence of SIRT2 increased acetylation levels of mitochondrial proteins [1]. Moreover, there is recent evidence that SIRT2 is critical for intact mitochondrial function, mitochondrial dynamics, and mitochondrial biogenesis [1, 29, 30]. Taken together, our results show that SIRT2 deletion, independent of diet and body weight, was sufficient to cause muscle insulin resistance. Our results are consistent with findings that illustrate the importance of maintaining the acetylation state in muscle mitochondria for insulin-stimulated muscle glucose metabolism [8, 26]. This is the first study to focus on SIRT2 *in vivo*, showing that muscle hyperacetylation is sufficient to impair insulin action in muscle of chow-fed mice.

The added challenge of HF-feeding was required for impairments in whole-body insulin action. The impairment in muscle R_g_ was further worsened in HF-fed SIRT2 KO mice, while muscle protein acetylation was not further augmented. This shows that muscle hyperacetylation by either diet or SIRT2 KO lead to similar levels of acetylation, but that these two processes are not additive. Our results show that while hyperacetylation in skeletal muscle is sufficient to impair muscle insulin sensitivity, HF-feeding is able to further blunt insulin action in SIRT2 KO mice independent of a further increase in total protein acetylation, whether cytosolic or mitochondrial. One cannot rule out that there is an increase in acetylation of a specific protein(s) that occurs without an increase in total protein acetylation. Interestingly, the vastus lateralis muscle did not exhibit significant defects in Rg. These differences between gastrocnemius and vastus lateralis muscles may be due to differences in susceptibility to protein acetylation imbalance, as the vastus lateralis muscle did not exhibit any changes in protein acetylation with either dietary or genetic manipulation.

Liver insulin resistance in response to HF diet is amplified by SIRT2 KO. The HF SIRT2 KO mice exhibited a significant reduction in insulin-induced suppression of endoR_a_. and SIRT2 KO mice on an HF diet had a ~8% greater body weight than WT mice. However, when the difference in body weight between WT and SIRT2 KO was eliminated by examining a subset of each genotype with overlapping body weights, the difference in endoRa was no longer observed. This indicates that the liver insulin resistance in HF-fed SIRT2 mice was attributable to increased body mass. Increases in body weight and/or fat mass have been shown to cause insulin resistance in multiple tissues, including liver and adipose tissue [31]. Liver inflammation was increased in the HF-fed SIRT2 KO mice, supporting an inhibitory role of SIRT2 on inflammation [32]. The present studies together with studies of others suggest that the observed reduction in SIRT2 protein in obese subjects may contribute to low-grade inflammation [28]. The present findings support the paradigm that weight gain, inflammation and insulin resistance are closely linked in liver and raise the possibility that the presence of SIRT2 may be protective to this process.

SIRT2 KO induced strong protein hyperacetylation in liver. This increase appeared to be driven specifically by SIRT2 deletion, and not by the increased body weight of the KO mice, as the mice assessed for protein acetylation did not have significantly different body weights. Moreover, while the protein acetylation profiles between WT and SIRT2 KO livers were strikingly different, the protein acetylation profile of chow and HF WT mice appeared qualitatively similar. HF-feeding induced liver protein hyperacetylation in the mitochondria, as shown previously [8, 12]. Protein acetylation was in contrast reduced in the liver cytosol of HF-fed WT mice. One major mechanism that could contribute to this is reduced cytosolic acetyl-CoA availability [33, 34]. This hypothesis is supported by the demonstration that acetyl-CoA is decreased in the livers of HF-fed mice [7]. The present studies show that SIRT2 is critical in restraining excessive protein acetylation in both liver and muscle. However, in contrast to the findings in muscle, liver hyperacetylation due to SIRT2 deletion in the absence of HF-feeding did not cause impaired hepatic insulin action. Our findings suggest that changes in energy balance (i.e the difference between energy intake and energy expenditure), as opposed to liver protein acetylation, may be the main driver for the liver insulin resistance observed in SIRT2 KO mice.

Our study identifies a strong role for SIRT2 in feeding behavior regarding highly palatable diets. Consistent with a previous report, we found that HF-fed SIRT2 KO mice exhibit an increase in weight gain [35]. We showed that this was due to a 40% increase in calorie intake in HF-fed SIRT2 KO mice. SIRT2 is the most abundant sirtuin in the brain, being highly expressed in nearly all brain cells [36, 37]. In particular, SIRT2 inhibitors have been shown to reverse anhedonia (i.e. lack of interest in a rewarding stimulus) and increase sucrose intake [38, 39]. This is consistent with our findings showing increased uptake of the high reward HF diet in the SIRT2 KO mice, and suggests a potential role for SIRT2 in satiety sensing in the face of high-calorie diets. This finding is further supported by the demonstration that SIRT2 downregulation is associated with decreased ATP levels in a neuronal cell line [40]. This would predictably lead to inhibition of hypothalamic proopiomelanocortin (POMC) anorexigenic neurons and activation of neuropeptide Y/agouti-related peptide (NPY/AgRP) orexigenic neurons via AMPK activation, which would promote feeding behavior [41]. In addition, SIRT2 is a critical negative regulator of adipogenesis, and this, along with overnutrition, could contribute to the increased fat mass observed in the HF-fed SIRT2 KO mice [42, 43].

In conclusion, the present study shows that whole body SIRT2 KO leads to widespread metabolic defects that include increased energy intake, adiposity, and insulin resistance in mice on a HF diet. We show that SIRT2 KO alone is sufficient to induce muscle insulin resistance in lean mice, and SIRT2 deletion exacerbates diet-induced insulin resistance, independent of a further increase in hyperacetylation. Although SIRT2 deletion is sufficient to induce muscle insulin resistance, this impairment is offset by effects on other tissues, so that it is undetectable by techniques used to study the whole body. We further show that liver insulin resistance in HF-fed mice is worsened by SIRT2 KO. This appears to be secondary to the increased fat mass due to increased caloric intake in HF SIRT2 KO mice. Consequently, the effects of SIRT2 on feeding behavior are a major driver of HF-feeding induced liver insulin resistance. These data show that SIRT2 protects against severe insulin resistance under conditions of overnutrition. These effects are particularly interesting since they are not associated with increases in total muscle or liver protein acetylation.

## Supporting information

S1 Fig HF diet does not alter SIRT2 protein in liver or muscle of WT mice. A. Immunoblot for SIRT2 in WT and SIRT2 KO soleus, gastrocnemius, vastus lateralis, liver, brain, and perigonadal white adipose tissue (WAT).
B. Immunoblot for SIRT2 on gastrocnemius (n = 6/group), vastus lateralis (n = 6/group), and liver (n = 8/group). Integrated intensities were obtained by the Odyssey software and normalized to GAPDH or β-actin. Black bars: chow; open bars: HF.
C, D: Relative intensities for SIRT3, normalized to GAPDH, in protein fractions extracted from gastrocnemius (C) or liver (D) from 5h-fasted WT and SIRT2 KO mice on either a chow of HF diet (n = 6/group).
E, F: Acetyl-CoA content was assessed in gastrocnemius and liver frozen tissue harvested from 5h fasted WT and SIRT2 KO mice on either a chow or HF diet. Two-way anova was performed followed by Tukey’s posthoc test. ## p<0.01 vs chow SIRT2 KO (n = 6/group).
G, H; Citrate synthase activity was assayed in homogenates from frozen gastrocnemius and liver tissues collected from 5h fasted WT and SIRT2 KO mice on either a chow or HF diet (n = 6/group).
(PDF)

S2 Fig Protein acetylation profile in whole brain of SIRT2 KO mice. Immunoblots for acetylated lysine on whole brain collected from 5h fasted WT and SIRT2 KO mice on a chow or HF diet. Integrated intensities of the individual bands were obtained from the 32 sec exposure image by the Odyssey software and normalized to GAPDH (n = 6/group). Two-way ANOVA was performed to determine significance.
(PDF)

S3 Fig IRS1 and mTOR phosphorylation in muscle of SIRT2 KO mice. Immunoblots for P-IRS1 (Ser302), total IRS1, P-mTOR (Ser2448) and total mTOR on whole gastrocnemius collected from insulin-clamped WT and SIRT2 KO mice on a chow or HF diet. Integrated intensities of the individual bands were obtained by the Odyssey software and normalized to their respective total protein (n = 6/group). Two-way ANOVA determined no statistical difference between groups.
(PDF)

S4 Fig Vastus lateralis protein acetylation was unchanged by diet or genotype. Relative intensities for acetylated lysine (AcK), normalized to GAPDH, in cytosolic (left) and mitochondrial (right) protein fractions extracted from vastus lateralis muscle from 5h-fasted WT and SIRT2 KO mice on either a chow of HF diet (n = 6/group).
(PDF)

S5 Fig Immunoblots for P-IRS1 (Ser302), IRS1, P-Akt (Ser473), Akt, P-FOXO1 (Ser256), FOXO1 and GAPDH on liver homogenates from clamped WT and SIRT2 KO mice. Quantification presented in Fig 6F–6H.
(PDF)

## References

[1] Liu G, Park SH, Imbesi M, Nathan WJ, Zou X, Zhu Y, et al Loss of NAD-Dependent Protein Deacetylase Sirtuin-2 Alters Mitochondrial Protein Acetylation and Dysregulates Mitophagy. Antioxid Redox Signal. 2017;26(15):849–63. 10.1089/ars.2016.6662 ; PubMed Central PMCID: PMCPMC5444513. 27460777 10.1089/ars.2016.6662 PMC5444513

[2] Gomes P, Outeiro TF, Cavadas C. Emerging Role of Sirtuin 2 in the Regulation of Mammalian Metabolism. Trends Pharmacol Sci. 2015;36(11):756–68. 10.1016/j.tips.2015.08.001 . 26538315 10.1016/j.tips.2015.08.001

[3] Yang L, Vaitheesvaran B, Hartil K, Robinson AJ, Hoopmann MR, Eng JK, et al The fasted/fed mouse metabolic acetylome: N6-acetylation differences suggest acetylation coordinates organ-specific fuel switching. J Proteome Res. 2011;10(9):4134–49. 10.1021/pr200313x ; PubMed Central PMCID: PMCPMC3204869. 21728379 10.1021/pr200313x PMC3204869

[4] Still AJ, Floyd BJ, Hebert AS, Bingman CA, Carson JJ, Gunderson DR, et al Quantification of mitochondrial acetylation dynamics highlights prominent sites of metabolic regulation. J Biol Chem. 2013;288(36):26209–19. 10.1074/jbc.M113.483396 ; PubMed Central PMCID: PMCPMC3764825. 23864654 10.1074/jbc.M113.483396 PMC3764825

[5] Wang Q, Zhang Y, Yang C, Xiong H, Lin Y, Yao J, et al Acetylation of metabolic enzymes coordinates carbon source utilization and metabolic flux. Science. 2010;327(5968):1004–7. 10.1126/science.1179687 ; PubMed Central PMCID: PMCPMC4183141. 20167787 10.1126/science.1179687 PMC4183141

[6] Zhao S, Xu W, Jiang W, Yu W, Lin Y, Zhang T, et al Regulation of cellular metabolism by protein lysine acetylation. Science. 2010;327(5968):1000–4. 10.1126/science.1179689 ; PubMed Central PMCID: PMCPMC3232675. 20167786 10.1126/science.1179689 PMC3232675

[7] Carrer A, Parris JL, Trefely S, Henry RA, Montgomery DC, Torres A, et al Impact of a High-fat Diet on Tissue Acyl-CoA and Histone Acetylation Levels. J Biol Chem. 2017;292(8):3312–22. 10.1074/jbc.M116.750620 ; PubMed Central PMCID: PMCPMC5336165. 28077572 10.1074/jbc.M116.750620 PMC5336165

[8] Davies MN, Kjalarsdottir L, Thompson JW, Dubois LG, Stevens RD, Ilkayeva OR, et al The Acetyl Group Buffering Action of Carnitine Acetyltransferase Offsets Macronutrient-Induced Lysine Acetylation of Mitochondrial Proteins. Cell Rep. 2016;14(2):243–54. 10.1016/j.celrep.2015.12.030 ; PubMed Central PMCID: PMCPMC4754083. 26748706 10.1016/j.celrep.2015.12.030 PMC4754083

[9] Lantier L, Williams AS, Williams IM, Yang KK, Bracy DP, Goelzer M, et al SIRT3 Is Crucial for Maintaining Skeletal Muscle Insulin Action and Protects Against Severe Insulin Resistance in High-Fat-Fed Mice. Diabetes. 2015;64(9):3081–92. 10.2337/db14-1810 ; PubMed Central PMCID: PMCPMC4542443. 25948682 10.2337/db14-1810 PMC4542443

[10] Wagner GR, Hirschey MD. Nonenzymatic protein acylation as a carbon stress regulated by sirtuin deacylases. Mol Cell. 2014;54(1):5–16. 10.1016/j.molcel.2014.03.027 ; PubMed Central PMCID: PMCPMC4040445. 24725594 10.1016/j.molcel.2014.03.027 PMC4040445

[11] Anderson KA, Hirschey MD. Mitochondrial protein acetylation regulates metabolism. Essays Biochem. 2012;52:23–35. 10.1042/bse0520023 ; PubMed Central PMCID: PMCPMC3872051. 22708561 10.1042/bse0520023 PMC3872051

[12] Pougovkina O, te Brinke H, Ofman R, van Cruchten AG, Kulik W, Wanders RJ, et al Mitochondrial protein acetylation is driven by acetyl-CoA from fatty acid oxidation. Hum Mol Genet. 2014;23(13):3513–22. 10.1093/hmg/ddu059 . 24516071 10.1093/hmg/ddu059

[13] Muoio DM, Noland RC, Kovalik JP, Seiler SE, Davies MN, DeBalsi KL, et al Muscle-specific deletion of carnitine acetyltransferase compromises glucose tolerance and metabolic flexibility. Cell Metab. 2012;15(5):764–77. 10.1016/j.cmet.2012.04.005 ; PubMed Central PMCID: PMCPMC3348515. 22560225 10.1016/j.cmet.2012.04.005 PMC3348515

[14] Kim HS, Vassilopoulos A, Wang RH, Lahusen T, Xiao Z, Xu X, et al SIRT2 maintains genome integrity and suppresses tumorigenesis through regulating APC/C activity. Cancer Cell. 2011;20(4):487–99. 10.1016/j.ccr.2011.09.004 ; PubMed Central PMCID: PMCPMC3199577. 22014574 10.1016/j.ccr.2011.09.004 PMC3199577

[15] Berglund ED, Li CY, Poffenberger G, Ayala JE, Fueger PT, Willis SE, et al Glucose metabolism in vivo in four commonly used inbred mouse strains. Diabetes. 2008;57(7):1790–9. 10.2337/db07-1615 ; PubMed Central PMCID: PMCPMC2453626. 18398139 10.2337/db07-1615 PMC2453626

[16] Ayala JE, Bracy DP, Julien BM, Rottman JN, Fueger PT, Wasserman DH. Chronic treatment with sildenafil improves energy balance and insulin action in high fat-fed conscious mice. Diabetes. 2007;56(4):1025–33. 10.2337/db06-0883 . 17229936 10.2337/db06-0883

[17] Steele R, Wall JS, De Bodo RC, Altszuler N. Measurement of size and turnover rate of body glucose pool by the isotope dilution method. Am J Physiol. 1956;187(1):15–24. 10.1152/ajplegacy.1956.187.1.15 . 13362583 10.1152/ajplegacy.1956.187.1.15

[18] Kraegen EW, James DE, Jenkins AB, Chisholm DJ. Dose-response curves for in vivo insulin sensitivity in individual tissues in rats. Am J Physiol. 1985;248(3 Pt 1):E353–62. 10.1152/ajpendo.1985.248.3.E353 . 3883806 10.1152/ajpendo.1985.248.3.E353

[19] Chan TM, Exton JH. A rapid method for the determination of glycogen content and radioactivity in small quantities of tissue or isolated hepatocytes. Anal Biochem. 1976;71(1):96–105. . 1275237 10.1016/0003-2697(76)90014-2

[20] Lighton JRB. Measuring metabolic rates: a manual for scientists. Oxford; New York: Oxford University Press; 2008 xiii, 201 p. p.

[21] Weir JB. New methods for calculating metabolic rate with special reference to protein metabolism. J Physiol. 1949;109(1–2):1–9. ; PubMed Central PMCID: PMCPMC1392602. 15394301 10.1113/jphysiol.1949.sp004363 PMC1392602

[22] Dimauro I, Pearson T, Caporossi D, Jackson MJ. A simple protocol for the subcellular fractionation of skeletal muscle cells and tissue. BMC Res Notes. 2012;5:513 10.1186/1756-0500-5-513 ; PubMed Central PMCID: PMCPMC3508861. 22994964 10.1186/1756-0500-5-513 PMC3508861

[23] Schmittgen TD, Livak KJ. Analyzing real-time PCR data by the comparative C(T) method. Nat Protoc. 2008;3(6):1101–8. . 18546601 10.1038/nprot.2008.73

[24] Kaiyala KJ. Mathematical model for the contribution of individual organs to non-zero y-intercepts in single and multi-compartment linear models of whole-body energy expenditure. PLoS One. 2014;9(7):e103301 10.1371/journal.pone.0103301 ; PubMed Central PMCID: PMCPMC4113365. 25068692 10.1371/journal.pone.0103301 PMC4113365

[25] Shoelson SE, Lee J, Goldfine AB. Inflammation and insulin resistance. J Clin Invest. 2006;116(7):1793–801. 10.1172/JCI29069 ; PubMed Central PMCID: PMCPMC1483173. 16823477 10.1172/JCI29069 PMC1483173

[26] Seiler SE, Koves TR, Gooding JR, Wong KE, Stevens RD, Ilkayeva OR, et al Carnitine Acetyltransferase Mitigates Metabolic Inertia and Muscle Fatigue during Exercise. Cell Metab. 2015;22(1):65–76. 10.1016/j.cmet.2015.06.003 ; PubMed Central PMCID: PMCPMC4754082. 26154055 10.1016/j.cmet.2015.06.003 PMC4754082

[27] LaBarge S, Migdal C, Schenk S. Is acetylation a metabolic rheostat that regulates skeletal muscle insulin action? Mol Cells. 2015;38(4):297–303. doi: 10.14348/molcells.2015.0020 ; PubMed Central PMCID: PMCPMC4400303. 25824547 10.14348/molcells.2015.0020 PMC4400303

[28] Krishnan J, Danzer C, Simka T, Ukropec J, Walter KM, Kumpf S, et al Dietary obesity-associated Hif1alpha activation in adipocytes restricts fatty acid oxidation and energy expenditure via suppression of the Sirt2-NAD+ system. Genes Dev. 2012;26(3):259–70. 10.1101/gad.180406.111 ; PubMed Central PMCID: PMCPMC3278893. 22302938 10.1101/gad.180406.111 PMC3278893

[29] Lemos V, de Oliveira RM, Naia L, Szego E, Ramos E, Pinho S, et al The NAD+-dependent deacetylase SIRT2 attenuates oxidative stress and mitochondrial dysfunction and improves insulin sensitivity in hepatocytes. Hum Mol Genet. 2017;26(21):4105–17. 10.1093/hmg/ddx298 . 28973648 10.1093/hmg/ddx298

[30] Silva DF, Esteves AR, Oliveira CR, Cardoso SM. Mitochondrial Metabolism Power SIRT2-Dependent Deficient Traffic Causing Alzheimer's-Disease Related Pathology. Mol Neurobiol. 2017;54(6):4021–40. 10.1007/s12035-016-9951-x . 27311773 10.1007/s12035-016-9951-x

[31] Kahn SE, Hull RL, Utzschneider KM. Mechanisms linking obesity to insulin resistance and type 2 diabetes. Nature. 2006;444(7121):840–6. 10.1038/nature05482 . 17167471 10.1038/nature05482

[32] Kim MJ, Kim DW, Park JH, Kim SJ, Lee CH, Yong JI, et al PEP-1-SIRT2 inhibits inflammatory response and oxidative stress-induced cell death via expression of antioxidant enzymes in murine macrophages. Free Radic Biol Med. 2013;63:432–45. 10.1016/j.freeradbiomed.2013.06.005 . 23770196 10.1016/j.freeradbiomed.2013.06.005

[33] Ghanta S, Grossmann RE, Brenner C. Mitochondrial protein acetylation as a cell-intrinsic, evolutionary driver of fat storage: chemical and metabolic logic of acetyl-lysine modifications. Crit Rev Biochem Mol Biol. 2013;48(6):561–74. 10.3109/10409238.2013.838204 ; PubMed Central PMCID: PMCPMC4113336. 24050258 10.3109/10409238.2013.838204 PMC4113336

[34] Wagner GR, Payne RM. Widespread and enzyme-independent Nepsilon-acetylation and Nepsilon-succinylation of proteins in the chemical conditions of the mitochondrial matrix. J Biol Chem. 2013;288(40):29036–45. 10.1074/jbc.M113.486753 ; PubMed Central PMCID: PMCPMC3790002. 23946487 10.1074/jbc.M113.486753 PMC3790002

[35] Bobrowska A, Donmez G, Weiss A, Guarente L, Bates G. SIRT2 ablation has no effect on tubulin acetylation in brain, cholesterol biosynthesis or the progression of Huntington's disease phenotypes in vivo. PLoS One. 2012;7(4):e34805 10.1371/journal.pone.0034805 ; PubMed Central PMCID: PMCPMC3325254. 22511966 10.1371/journal.pone.0034805 PMC3325254

[36] Maxwell MM, Tomkinson EM, Nobles J, Wizeman JW, Amore AM, Quinti L, et al The Sirtuin 2 microtubule deacetylase is an abundant neuronal protein that accumulates in the aging CNS. Hum Mol Genet. 2011;20(20):3986–96. 10.1093/hmg/ddr326 ; PubMed Central PMCID: PMCPMC3203628. 21791548 10.1093/hmg/ddr326 PMC3203628

[37] Zhu H, Zhao L, Wang E, Dimova N, Liu G, Feng Y, et al The QKI-PLP pathway controls SIRT2 abundance in CNS myelin. Glia. 2012;60(1):69–82. 10.1002/glia.21248 ; PubMed Central PMCID: PMCPMC3217119. 21948283 10.1002/glia.21248 PMC3217119

[38] Liu R, Dang W, Du Y, Zhou Q, Jiao K, Liu Z. SIRT2 is involved in the modulation of depressive behaviors. Sci Rep. 2015;5:8415 10.1038/srep08415 ; PubMed Central PMCID: PMCPMC4325337. 25672834 10.1038/srep08415 PMC4325337

[39] Munoz-Cobo I, Belloch FB, Diaz-Perdigon T, Puerta E, Tordera RM. SIRT2 inhibition reverses anhedonia in the VGLUT1+/- depression model. Behav Brain Res. 2017;335:128–31. 10.1016/j.bbr.2017.07.045 . 28778545 10.1016/j.bbr.2017.07.045

[40] Nie H, Chen H, Han J, Hong Y, Ma Y, Xia W, et al Silencing of SIRT2 induces cell death and a decrease in the intracellular ATP level of PC12 cells. Int J Physiol Pathophysiol Pharmacol. 2011;3(1):65–70. ; PubMed Central PMCID: PMCPMC3068855. 21479103 PMC3068855

[41] Oh TS, Cho H, Cho JH, Yu SW, Kim EK. Hypothalamic AMPK-induced autophagy increases food intake by regulating NPY and POMC expression. Autophagy. 2016;12(11):2009–25. 10.1080/15548627.2016.1215382 ; PubMed Central PMCID: PMCPMC5103348. 27533078 10.1080/15548627.2016.1215382 PMC5103348

[42] Wang F, Tong Q. SIRT2 suppresses adipocyte differentiation by deacetylating FOXO1 and enhancing FOXO1's repressive interaction with PPARgamma. Mol Biol Cell. 2009;20(3):801–8. 10.1091/mbc.E08-06-0647 ; PubMed Central PMCID: PMCPMC2633403. 19037106 10.1091/mbc.E08-06-0647 PMC2633403

[43] Jing E, Gesta S, Kahn CR. SIRT2 regulates adipocyte differentiation through FoxO1 acetylation/deacetylation. Cell Metab. 2007;6(2):105–14. 10.1016/j.cmet.2007.07.003 ; PubMed Central PMCID: PMCPMC2083635. 17681146 10.1016/j.cmet.2007.07.003 PMC2083635

