# Supplementary figures and images for: SIRT2 knockout exacerbates insulin resistance in high fat-fed mice
Source: PLoS One. 2018 Dec 11;13(12):e0208634. doi: 10.1371/journal.pone.0208634 (PMC6289500; doi:10.1371/journal.pone.0208634)

**Figure S1**

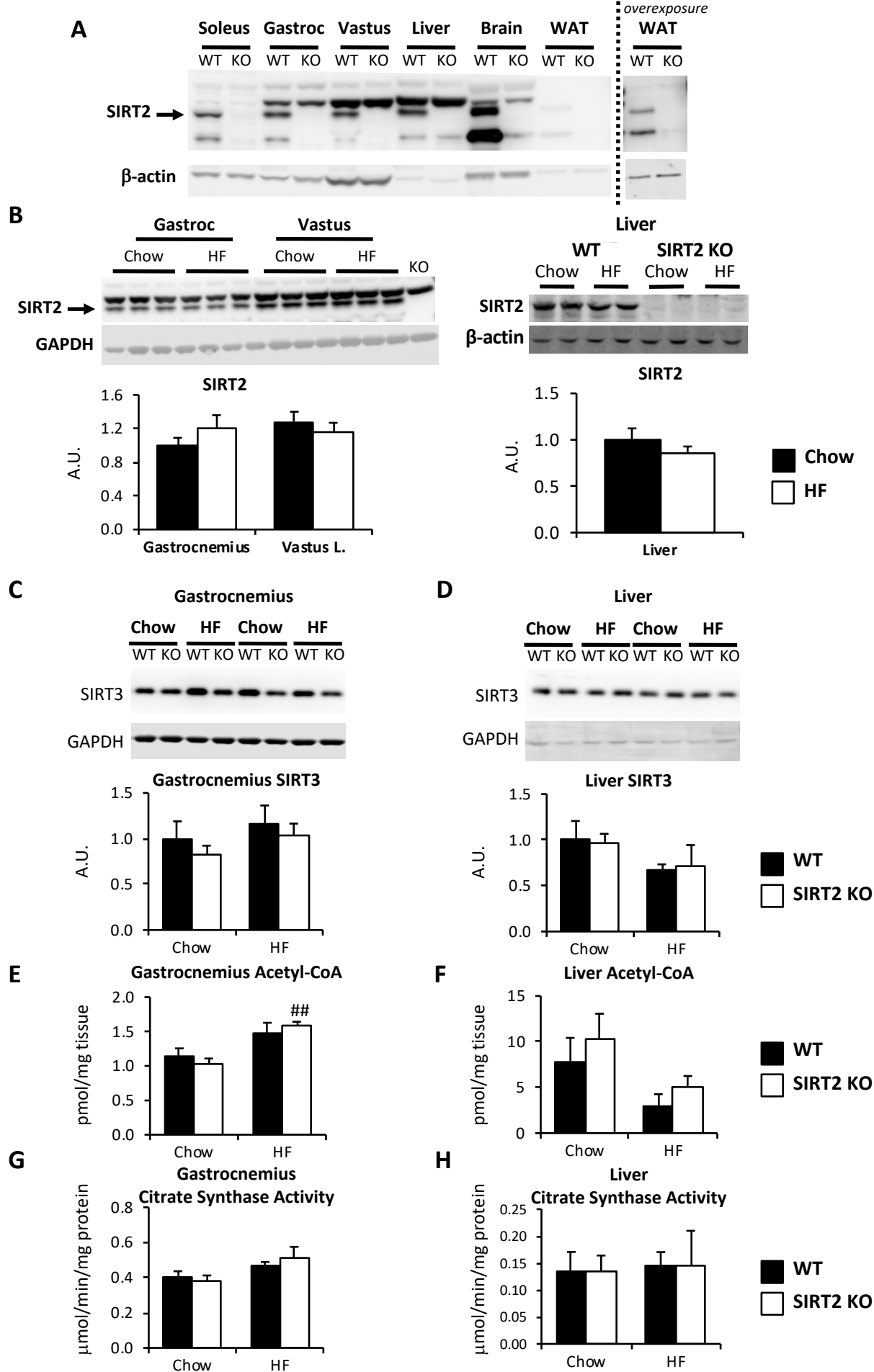

Supplement: S1 Fig — A. Immunoblot for SIRT2 in WT and SIRT2 KO soleus, gastrocnemius, vastus lateralis, liver, brain, and perigonadal white adipose tissue (WAT). B. Immunoblot for SIRT2 on gastrocnemius (n = 6/group), vastus lateralis (n = 6/group), and liver (n = 8/group). Integrated intensities were obtained by the Odyssey software and normalized to GAPDH or β-actin. Black bars: chow; open bars: HF. C, D: Relative intensities for SIRT3, normalized to GAPDH, in protein fractions extracted from gastrocnemius (C) or liver (D) from 5h-fasted WT and SIRT2 KO mice on either a chow of HF diet (n = 6/group). E, F: Acetyl-CoA content was assessed in gastrocnemius and liver frozen tissue harvested from 5h fasted WT and SIRT2 KO mice on either a chow or HF diet. Two-way anova was performed followed by Tukey’s posthoc test. ## p<0.01 vs chow SIRT2 KO (n = 6/group). G, H; Citrate synthase activity was assayed in homogenates from frozen gastrocnemius and liver tissues collected from 5h fasted WT and SIRT2 KO mice on either a chow or HF diet (n = 6/group). (PDF) [file pone.0208634.s001.pdf]

**Figure S2**

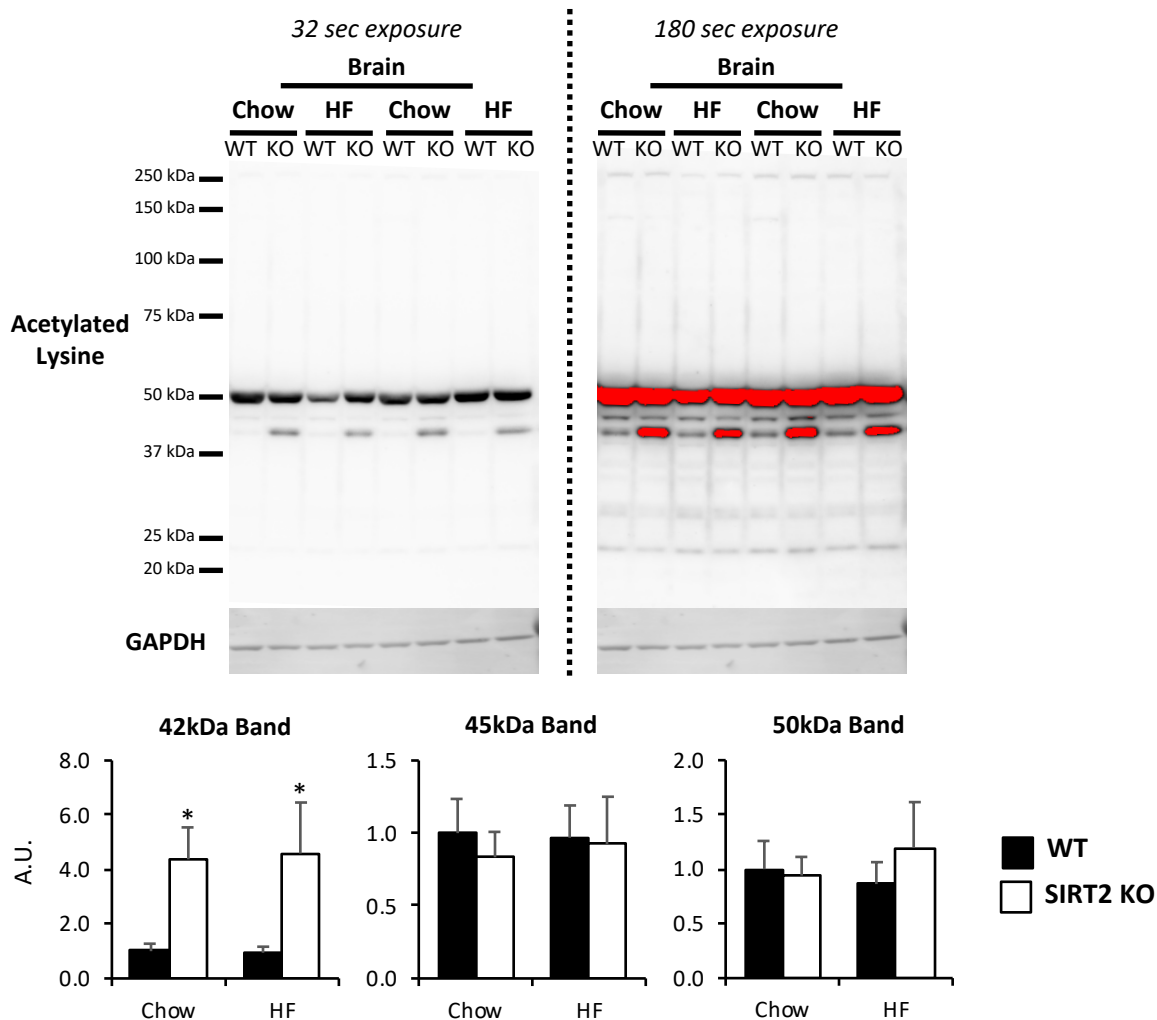

Supplement: S2 Fig — Immunoblots for acetylated lysine on whole brain collected from 5h fasted WT and SIRT2 KO mice on a chow or HF diet. Integrated intensities of the individual bands were obtained from the 32 sec exposure image by the Odyssey software and normalized to GAPDH (n = 6/group). Two-way ANOVA was performed to determine significance. (PDF) [file pone.0208634.s002.pdf]

**Figure S3**

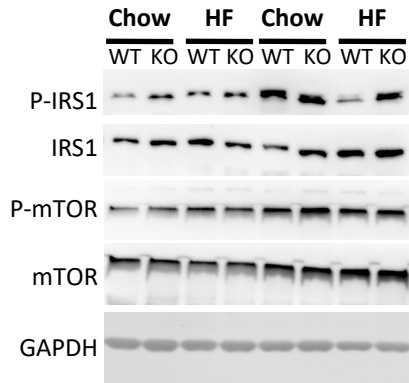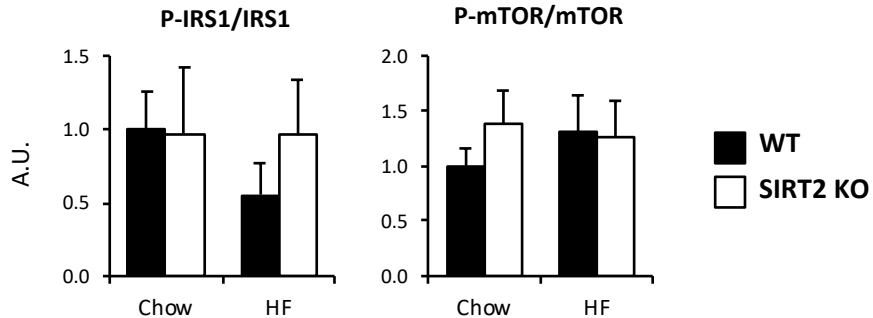

Supplement: S3 Fig — Immunoblots for P-IRS1 (Ser302), total IRS1, P-mTOR (Ser2448) and total mTOR on whole gastrocnemius collected from insulin-clamped WT and SIRT2 KO mice on a chow or HF diet. Integrated intensities of the individual bands were obtained by the Odyssey software and normalized to their respective total protein (n = 6/group). Two-way ANOVA determined no statistical difference between groups. (PDF) [file pone.0208634.s003.pdf]

**Figure S4**

**Vastus Lateralis**

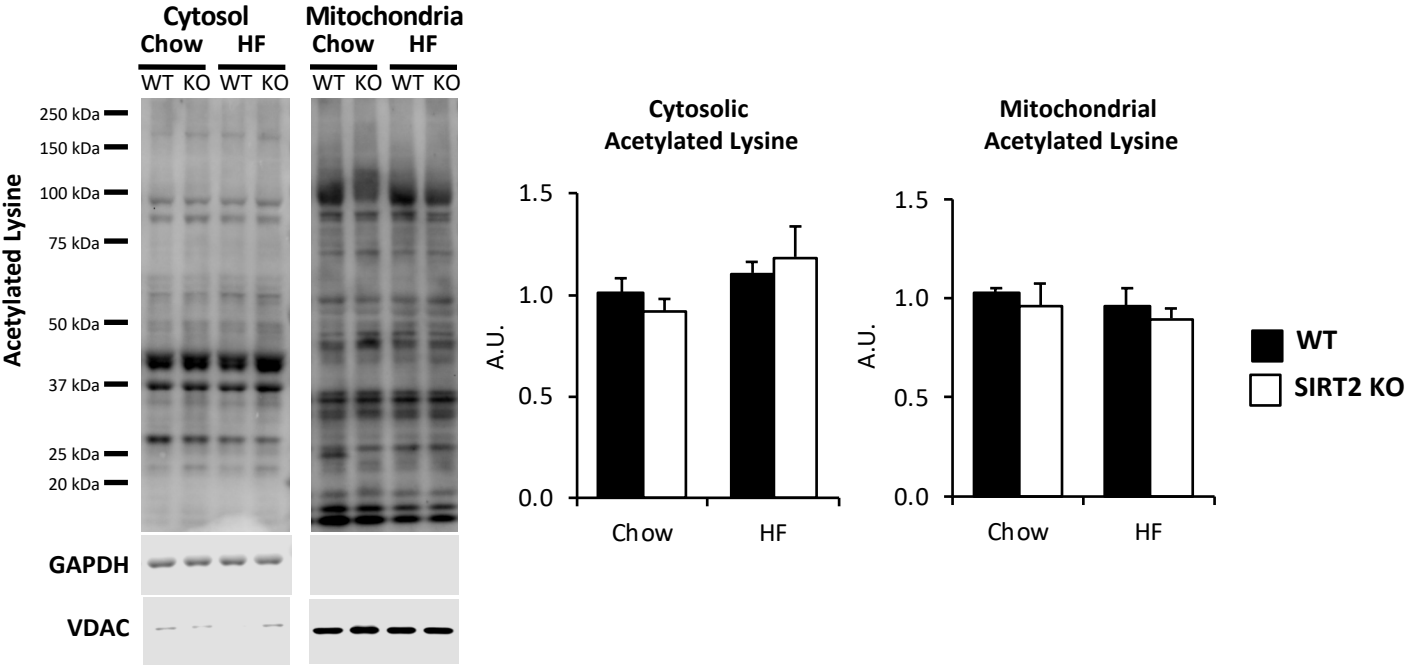

Supplement: S4 Fig — Relative intensities for acetylated lysine (AcK), normalized to GAPDH, in cytosolic (left) and mitochondrial (right) protein fractions extracted from vastus lateralis muscle from 5h-fasted WT and SIRT2 KO mice on either a chow of HF diet (n = 6/group). (PDF) [file pone.0208634.s004.pdf]

**Figure S5**

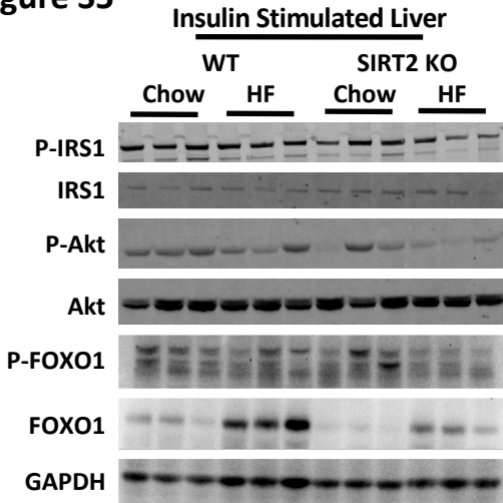

Supplement: S5 Fig — Quantification presented in Fig 6F–6H. (PDF) [file pone.0208634.s005.pdf]
